# Influence of microcirculation load on FFR in coronary artery stenosis model

**DOI:** 10.1186/s12872-020-01437-w

**Published:** 2020-03-21

**Authors:** Hongzeng Xu, Jing Liu, Donghui Zhou, Yuanzhe Jin

**Affiliations:** Department of Cardiology, The fourth Affiliated Hospital of China Medical University, No. 4, Chongshan Road, Huanggu District, Shenyang, 110032 China

**Keywords:** Coronary artery disease, Microcirculation, Hemodynamics, FFR

## Abstract

**Background:**

The coronary artery hemodynamics are impacted by both the macrocirculation and microcirculation. Whether microcirculation load impact the functional assessment of a coronary artery stenosis is unknown. The purpose of this study is to investigate the effect of porous media of the microcirculation on fractional flow reserve (FFR) in stenotic coronary artery model.

**Methods:**

A three dimensional computational simulation of blood flow in coronary artery symmetric stenotic model was constructed. The computational fluid dynamics (CFD) model was developed with Fluent 16.0. Blood was modeled as a shear thinning, non-Newtonian fluid with the Carreau model. A seepage outlet boundary condition and transient inlet conditions were imposed on the model. Coronary physiologica diagnostic parameter such as pressure, velocity and fractional flow reserve (FFR) were investigated in the model and compared with the microcirculation load (ML) and constant pressure load (PL) condition.

**Results:**

The present study showed the different hemodynamics in the ML and PL condition. The pre-stenotic pressure is almost the same in the two model. However the pressure in the post-stenotic artery domain is much lower in the PL model. The fluctuation range of the pressures is much higher in ML model than those in PL model. The velocity flow was more steady and lower in the ML model. For the PL model with 75% artery stenosis the FFR was 0.776, while for the ML model with the same stenosis, the FFR was 0.813.

**Conclusions:**

This study provides evidence that FFR increased in the presentation of ML condition. There is a strong hemodynamic effect of microcirculation on coronary artery stenosis.

## Background

Coronary artery disease (CAD) are characterized with epicardial stenosis and microcirculation dysfunction. And the coronary artery hemodynamics are affected both by the epicardial stenotic coronary artery impede and microcirculation load. Regulation of coronary blood flow is quite complex [1], epicardial stenosis severity and microcirculatory resistance may affect each other [2, 3]. However, the complex interrelationship between the coronary microcirculation and the epicardial coronary arteries contributing to the coronary artery hemodynamics remains poorly understood and is controversial [4]. Some recent studies have suggested that microvascular resistance at maximal vasodilation will increase when the severity of epicardial disease increases [5]. While other studies showed that coronary microcirculatory resistance is independent of epicardial coronary artery stenosis [6], and coronary microcirculatory resistance is not influenced by the epicardial stenosis severity [7]. Now, epicardial coronary artery stenosis is regarded to exert their pathological role mainly through a limitation on maximal flow capacity in the distal vascular bed. However, how the distal microcirculation bed impact the hemodynamics of coronary artery was unknown. It’s believed that the microcirculation load has a great influence on the bloodstream, and has important influence on various phenomena in the flow field [8]. Moreover, such microvascular alterations with altered microvascular resistance may partly obscure Fractional flow reserve (FFR) measurements [9, 10]. Therefore, a complete hemodynamic model is needed to take into account the influence of microcirculation load effect.

Computational fluid dynamics (CFD) simulation has been widely used to study the hemodynamic parameters in coronary arteries due to the limitation of in vivo measurements [11]. FFR is currently used as a gold standard for the assessment of functional significance of stenosis severity and is applied for guiding cardiovascular intervention. Recently, the CFD approach has been used to determine the FFR from the clinical medical image or numerical simulation. In these CFD hemodynamics simulations, boundary conditions are vital for obtaining accurate flow patterns. Hewever, there are lack of numerical simulation studies to determine FFR and hemodynamic changes due to microcirculation impedance outlet boundary conditions.

The resistance offered by microcirculation to blood flow can be conceived as the resistance encountered by fluid passing through porous medium [12]. It has been established that the flow in microcirculation can be simulated by using a porous flow model [13, 14]. Based on seepage theory [15], the seepage flow could be specified as a boundary condition which shows that the resistance caused by the seepage condition in microcirculation. To obtain the large and complex microvascular anatomy is difficult, a porous medium model may replicate the downstream capillary structures, which can supply the essential characteristics of the seepage flow in microcirculation. Therefore, a load of porous media seepage could be imposed at the outlet of the artery stenosis model to simulate the microcirculation. In this study, a 3D computational coronary stenotic artery model with outlet condition of microcirculation load (ML) and constant pressure load (PL) was constructed. The purpose of this study is to investigate the effect of porous media of the microcirculation on coronary artery stenosis hemodynamics.

## Methods

### Geometry model

**Fig. 1:**
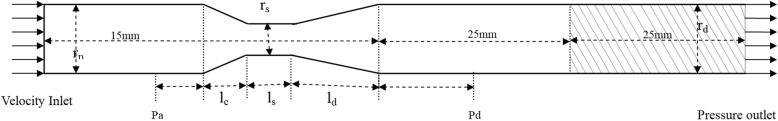
Schematic diagram showing artery stenosis and a seepage outlet boundary condition for hemodynamics applications

In this work, the artery model was considered as a symmetric stenotic geometry as shown in Fig. 1. Stenotic regions consist of converging (of length l_c_ = 1.5 mm), throat (of radius r_s_ = 0.7 mm and length l_s_ = 1.2 mm) and diverging (of length l_d_ = 3 mm) sections which was considered as trapezoidal. Moreover, proximal and distal radii are assumed to be identical (r_p_ = r_d_ = 1.4 mm). Artery area stenosis (AS) percentage was 75%, which was defined as:

1 $$ \frac{r_p^2-{r}_s^2}{r_p^2}\times 100\% $$

### Computational blood flow model

It was assumed that the flow of blood in the coronary artery is incompressible and governed by the Navier-Stokes equations

2 $$ \rho \left(\frac{\partial_u}{\partial_t}+u\cdot \nabla u\right)=-\nabla p+\nabla \cdot T $$

and the continuity equation for incompressible flow is

3 $$ \nabla \cdot u=0 $$

Here *u* is the three dimensional velocity vector, t the time, *ρ* the blood density, *p* the pressure and *T* the stress tensor. The flow was set as laminar with a density of 1060 kg/m^3^_._

As is well known, the viscoelasticity and shear thinning of the blood [16] are closely relevant to its microscopic features, such as deformation, aggregation and alignment of the red blood cells. In this study, the blood flow was modeled to be homogenous and non-Newtonian, and we used the Carreau model

4 $$ \mu ={\mu}_{\infty }+\left({\mu}_0-{\mu}_{\infty}\right){\left[1+{\left(\lambda \gamma \right)}^2\right]}^{\left(n-1\right)/2} $$

where μ is the dynamic viscosity, μ_0_ and μ_∞_ are the viscosity as the shear rate goes to infinity and zero, γ is the shear rate, λ is the time constant and n is the power-law index. All values were taken from the literature, μ_0_ = 0.0560 Pa·S, μ_∞_ = 0.00345 Pa·S, λ = 3.313 s and *n* = 0.3568 [17].

### Microcirculation domain

Accounting the aspect of circulation system structure, a complete hemodynamic model requires consideration of the microcirculation load effect. Microcirculation is considered as a porous medium. In this study, the outlet impedance, as a seepage boundary condition, is provided by the flow in the microcirculation porous zone. We set the porosity of the microcirculation to *φ* = 0.5 [18]. The following empirical equation is adopted to compute the microcirculation zone permeability, which is determined by the porosity:

5 $$ \mathrm{k}=\frac{d^2{\varphi}^3}{180{\left(1-\varphi \right)}^2} $$

where d is the diameter of the microvessel, and here we set d = 100 μm following the literature [12]. The governing equations in the microcirculation zone are determined as follows:

6 $$ \rho \left(\frac{\partial_{\left(\phi u\right)}}{\partial_t}+u\cdot \nabla \left(\phi u\right)\right)=-\nabla \left(\phi p\right)+\nabla \cdot \left(\phi T\right)-\frac{\phi^2\eta }{k}u $$

7 $$ \nabla \cdot u=0 $$

### Boundary conditions and microcirculation domain

**Fig. 2:**
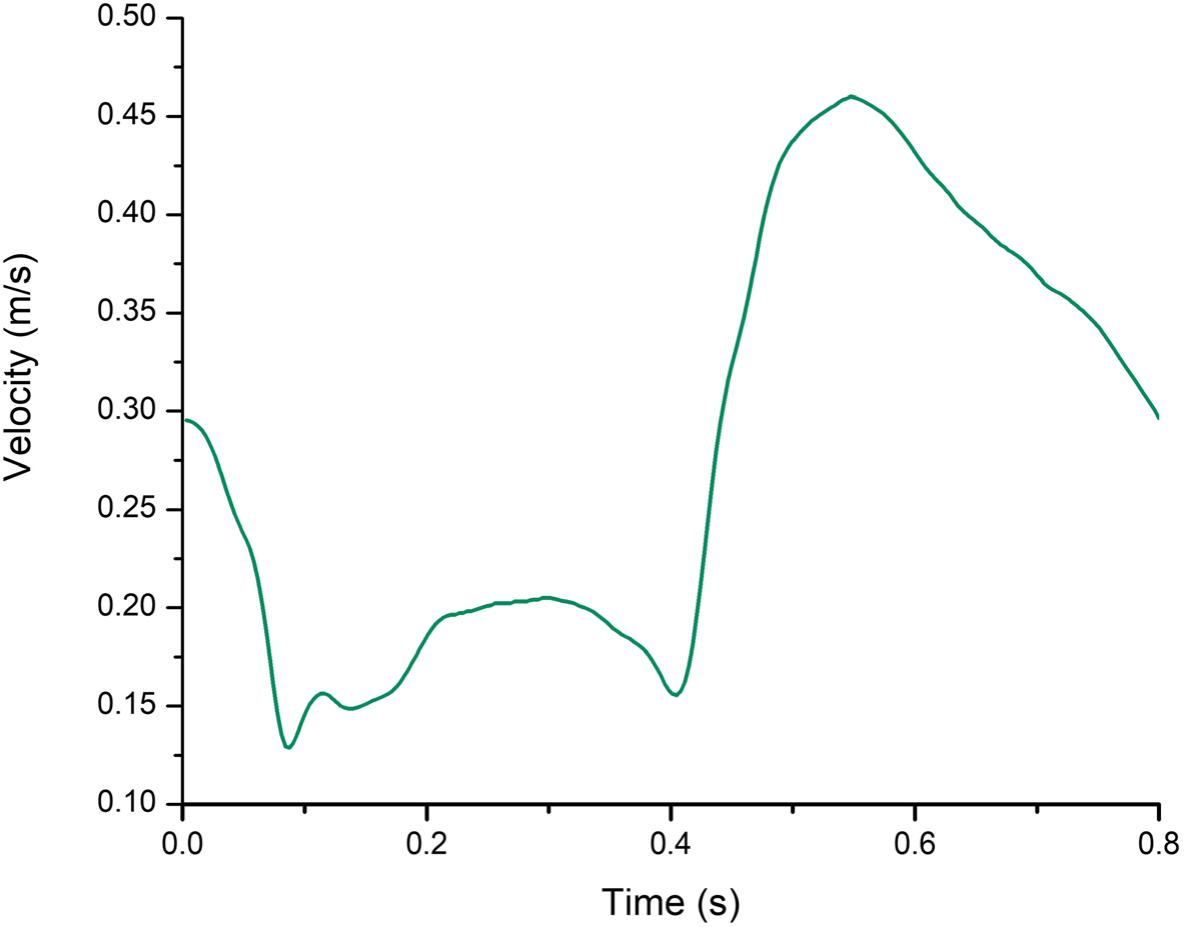
The inlet velocity profile

As shown in Fig. 2, a transient time-dependent velocity profile of a typical coronary artery was applied at the inlet [19]. The maximum velocity was 0.46 m/s, the average velocity was 0.28 m/s and the minimum velocity was 0.13 m/s. Accordingly, in the example of normal physiology, the heart rate was 75/min and the period of the flow waveform is 0.8 s. Both ML and PL cases were solved with the same inlet boundary conditions. The outlet condition was pressure free, and was set as the distal venules pressure 3333 Pascal (Pa, 25 mmHg) for the PL case [20, 21] and 0 Pa for the ML case. While in the ML cases the microcirculation zone is coupled at the outlet. We assume that the vessel walls are rigid and all velocity components were set to zero at the wall according to no-slip condition.

### Numerical methodology

The computational domains were initially meshed with hexahedral and tetrahedral elements. The total number of elements vary from 200,000 to 295,000 for both ML and PL models. Computational simulations were performed with Fluent 16.0 (Ansys Inc., Canonsburg, USA). The transient flow analysis was run for 247 time steps (0.0032 s per time step) representing pulsatile flow with each time step converging to a residual target of 1 × 10^− 5^.

### FFR calculation

FFR is the well-studied physiological parameter to guide coronary revascularization decision in clinical practice. FFR is defined as the approximate ratio of distal coronary pressure to aortic pressure [22].

8 $$ \mathrm{FF}R=\frac{{\overset{\sim }{\mathrm{P}}}_d-{\mathrm{P}}_v}{{\overset{\sim }{\mathrm{P}}}_a-{\mathrm{P}}_v} $$

where ${\overset{\sim }{\mathrm{P}}}_a$ is defined as the time averaged aortic pressure, ${\overset{\sim }{\mathrm{P}}}_d$ is the time averaged distal stenotic pressure measured at the end of flow reversal occurring and P_v_ is the venous pressure which is assumed to be 0 mmHg.

## Results

In both ML and PL models, the pressure and velocity distribution was simulated and FFR was analyzed.

### Pressures profile at different models

**Fig. 3:**
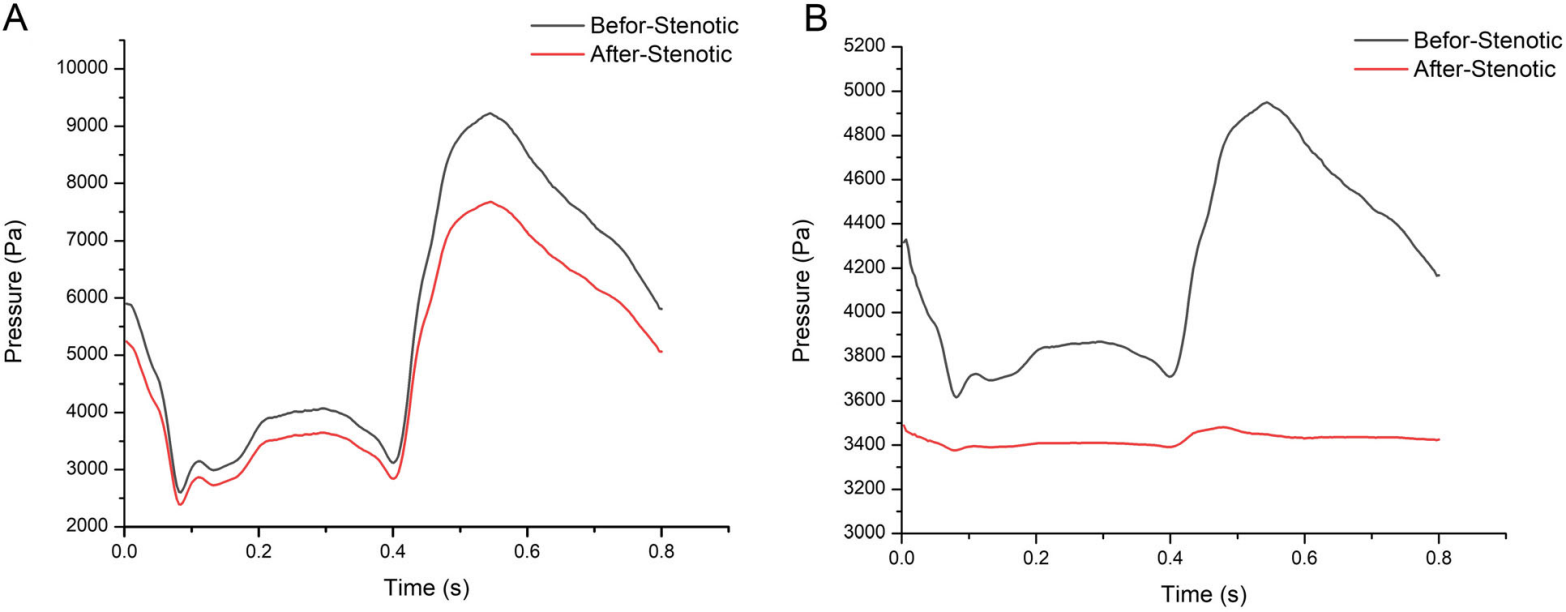
Pressure profiles in the artery zone**. a** Pressure profiles in the ML model. **b** Pressure profiles in the PL model

Fig. 3 depicts the pressure distributions before and after stenosis of the artery zone under the microcirculation seepage and pressure outlet boundary condition. The pressure profile at the both sides of the stenosis at the artery zone are almost identical with the ML and PL conditions. The average of pre-stenotic pressure (${\overset{\sim }{\mathrm{P}}}_a$ =4331 Pa) and post-stenotic pressure (${\overset{\sim }{\mathrm{P}}}_d$ =3522 Pa) in the ML model were higher than those (${\overset{\sim }{\mathrm{P}}}_a$ = 3611 Pa, ${\overset{\sim }{\mathrm{P}}}_d$ = 2805 Pa) in the PL model.

As shown in Table 1, a large pressure drop occurs across the stenosis. The absolute values of the pressure drops across the stenosis were similar in the two models. However, the fluctuation range of the pressures varied with different boundary conditions. In the ML model, the fluctuation range of the pressures were higher than those in PL model.

**Table 1:** The pressure difference in the artery zone and FFR calculation under the two outlet boundary conditions

Outlet	The peak Pressure (Pa)		The low pressure (Pa)		Fluctuation range (Pa)		FFR	AS(%)
	P_a_	P_d_	P_a_	P_d_	P_a_ (%)	P_d_ (%)		
Microcirculation Seepage Load	7170	5480	1980	1760	5190 (72.4%)	3720 (67.9%)	0.813	75%
Pressure Load	4540	2870	2960	2740	1580 (34.8%)	130 (4.53%)	0.776	75%

*FFR* Fractional flow researve, *Pa* Pressure at the aortic stenosis, *Pd* Pressure distal to the coronary stenosis, *AS* Area stenosis

### Velocity profile at different models

**Fig. 4:**
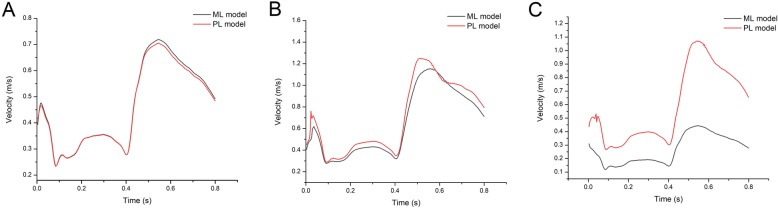
Velocity profiles in the artery zone**. a** Velocity profiles in the Pre-stenotic domain. **b** Velocity profiles in the Post-stenotic domain. **c** Velocity profiles in the microcirculation domain

Fig. 4 plots the different velocities in the two models of the artery zone. The time-dependent velocities in the artery zone with ML and PL condition are similar and resemble the velocity profiles imposed on the inlet. It shows that velocities in post-stenotic domain are more larger than the pre-stenotic domain in the two model because of the artery stenosis effect. While in the post-stenotic artery zone, it is found that the velocities in the PL model are higher than those in the ML model. Clearly, the flow is impeded by the artery stenosis so that high flow velocities are produced to maintain the blood supply.

As shown in Fig. 4, the velocity distributions in the microcirculation zone are similar to those in other artery zone. However, the velocities in the microcirculation zone in ML model are much lower than the PL condition model. We know that the diameter of the microvessels is less than 300 μm [23], so the corresponding velocities should be very low. In the ML model the waveform of the blood flow is more regular than that in the PL model, maybe the microcirculation porous media has a filtering effect on the circulation. Although the blood flow entering from the larger arteries is pulsatile, the flow in microcirculation is tend to be steady because of the repelling the pulsations by the capillaries.

### FFR calculation

After obtained the averaged pressure drop $\varDelta \overset{\sim }{\mathrm{P}}$ and the averaged pressure proximal to stenosis ${\overset{\sim }{\mathrm{P}}}_a$ from our computational study, FFR (= ${\overset{\sim }{\mathrm{P}}}_d$ / ${\overset{\sim }{\mathrm{P}}}_a$ = 1- $\varDelta \overset{\sim }{\mathrm{P}}$ / ${\overset{\sim }{\mathrm{P}}}_a$) was calculated. The FFR for the PL model was 0.776, while the FFR for the ML model was 0.813. The results showed that with the presence of microvasculature resistance, blood flow through the fixed stenosis will decrease, and FFR will increase. So the FFR value was impact obviously by the microcirculation upload condition.

## Discussion

In the present study, we constructed the artery stenosis model with ML and PL conditions. Porous medium modeling was used for analyzing biomass transport phenomena across within biological tissues, and we study the impact of microcirculation load on the pressure and flow field and hence the diagnosis parameter FFR of the artery stenosis model. It can be demonstrated that the downstream microvascular impedance of porous media which represents the flow resistance imposed by microcirculation, plays a dominant role compared with the stenosis local resistance. And the results showed that microcirculation load has significant effect on the coronary artery FFR measurement.

### The impact of microcirculation on blood hemodynamics

In this study, the fluctuation range of the pressures in the ML model was larger than in the PL model, which is more prone to a physiological fact. The results show that in coronary stenotic artery model the microvascular system seems to have an essential role in regulating blood pressure. In normal arterial structure, microvessels usually supply most of the resistance to blood flow so that the pressure wave propagation is impeded and the reflection is produced [24]. The constitutive equation of microcirculation load has a decisive influence on various phenomena in the flow field. During systole period, blood which is not discharged in time is stored in the elastic artery. After entering the diastole period, the fluid stored will gradually flow out of the artery. Microcirculation is very important in circulation and serves a function to reallocate blood flow from a time scale. Therefore, in hemodynamic numerical simulation study, the influence of microcirculation load must be considered.

### The impact of microcirculation on FFR

FFR is a well-validated clinical parameter derived from the measurement of coronary pressures and has greatly changed revascularization decision in clinical practice worldwide [25]. And now FFR is regarded as a gold-standard test for myocardial ischemia. Although FFR-guided percutaneous coronary intervention (PCI) in stable CAD could reduce the rate of myocardial infarction or urgent revascularization, there is a debate on whether FFR-guided PCI in stable CAD could reduce the rate of all-cause mortality. The recent Parikh’s study showed that the FFR-guided PCI reduce the the mortality compared with angiography-only guided PCI at 1 year follow-up [26]. However, after the 2 year follow-up, the FFR-guided PCI plus optimal medical therapy versus optimal medical therapy study showed a limited power of FFR to identify stenosis that require revascularization to prevent adverse events [27], whereas stenosis judged nonsignificant by FFR are still prone to adverse cardiac events. And another meta-analysis included individual patient data of three available randomized trials of contemporary FFR-guided PCI versus medical therapy for patients with stable coronary lesions and find that there was little evidence for a difference between groups in cardiac or all-cause deaths [28].

It should be noted that FFR is determined under the hypothesis of minimal and absolute stable microvascular resistance. The intravenous administration of adenosine is assumed to lead to a minimal and stable magnitude of coronary resistance in most patients. However, it is likely unattainable in the clinical practice. Microvascular resistance is unstable and variable even at maximal vasodilation condition [29]. The vasodilation mediator adenosine is far from able to abolish all vasoconstrictor tone [30]. In reality, adenosine only abolishes part of the coronary vasomotor tone in the individual patient. So it is speculated that the microcirculation coundn’t be neglected in the simulation of coronary artery hemodynamics.

Coronary autoregulation is a multifactorial and complex process regulated by many mediators. And the functional loss of microcirculation will cause functional stenosis. A specific constant pressure profile is usually imposed at the outlet in traditional computational hemodynamics simulation. However, free outlet and constant load outlet do not meet the physiological requirements, and microcirculation load outlet condition can better simulate the physiology situation. In our study, the porous media of the microcirculation is shown to play an important role in the FFR calculation.

### The structure and function of microcirculation

Coronary microvascular networks play a key role in determining blood flow in the heart. Alterations in the coronary microvasculature may exclusively determine the development of myocardial ischaemia [31]. Nellis et al. demonstrated that arteriolar and venular vessels smaller than 140 μm in diameter contributed 70% of coronary vascular resistance in the ventricle. And Chilian et al. study the left ventricle and found that more than 50% of total coronary resistance arterioles less than 150 μm in diameter [32].

As an important part of the arterial circulation, microvasculature plays important roles in many aspects, such as interstitial fluid generation, substance exchange and inverse flow. And the flow fields in the microcirculation zone may determine these processes. In this study, microvasculature is treated as a porous medium. When the porosity of the microcirculation zone are changed, the flow fields would be disturbed. Many disease such as diabetes, hypertension and hyperlipemia may lead to such microcirculation porosity changes. Study the hemodynamic changes induced by the pathological conditions may help us develop drugs treatment strategies (such as ACEI) which improved the microcirculation.

### Study limitations and future directions

The present study has several limitations. Firstly, an ideal cylinder model was constructed rather than the patient-specific case. Clinically, the artery of the patient may be bifurcated and highly tortuous, and the haemodynamic research of that may show different outcomes. In addition, there is a fluid-structure interaction (FSI) and a seepage boundary condition between blood and arterial wall. This study didn’t included the FSI effect. Thus in future work, a more severe case may be constructed included FSI study in association with the model using a seepage boundary condition. Thirdly, factors that influence the hemodynamic parameters such as vessel bending due to heart motion, lesion curvature and wall roughness were not included in this study. Using the data from the clinical and further experimental study would validate the computational results in the future as well.

## Conclusions

Myocardial ischaemia may be caused by irregularities of both epicardial arteries and downstream microvascular networks. It was found that FFR value was slightly decreased with the presence of microcirculation load.

## Abbreviations

FFR: Fractional flow reserve
CFD: Computational fluid dynamics
CAD: Coronary artery disease
PCI: Percutaneous coronary intervention
ML: Microcirculation load
PL: Pressure load
FSI: Fluid-structure interaction
AS: Area stenosis
Pa: Pressure at the aortic stenosis
Pd: Pressure distal to the coronary stenosis
